# Doomsday Prepping During the COVID-19 Pandemic

**DOI:** 10.3389/fpsyg.2021.659925

**Published:** 2021-04-14

**Authors:** Nina Smith, Susan Jennifer Thomas

**Affiliations:** ^1^Faculty of Social Sciences, School of Psychology, University of Wollongong, Wollongong, NSW, Australia; ^2^Illawarra Health and Medical Research Institute, University of Wollongong, Wollongong, NSW, Australia; ^3^Faculty of Science, Medicine and Health, University of Wollongong, Wollongong, NSW, Australia

**Keywords:** doomsday prepping, anxiety, OCD, masculinity, hoarding, pandemic (COVID-19)

## Abstract

“Doomsday prepping” is a phenomenon which involves preparing for feared societal collapse by stockpiling resources and readying for self-sufficiency. While doomsday prepping has traditionally been reported in the context of extremists, during the COVID-19 pandemic, excessive stockpiling leading to supply shortages has been reported globally. It is unclear what psychological or demographic factors are associated with this stockpiling. This study investigated doomsday prepping beliefs and behaviors in relation to COVID-19 proximity, demographics, coping strategies, psychopathology, intolerance of uncertainty (IU), and personality in 384 participants (249 female) in an online study. Participants completed a number of questionnaires including the Post-Apocalyptic and Doomsday Prepping Beliefs Scale and a scale designed for the current study to measure prepping in the context of COVID-19. These were analyzed using ANOVAs, correlational, and mediation analyses to examine relationships between psychometric variables and stockpiling. Prepping beliefs and behaviors were higher in males than females and positively associated with anxiety, obsessive-compulsive symptoms, IU, and traditional masculinity traits. Older age, male gender, obsessive-compulsive symptoms, and traditional masculinity predicted unique variance in prepping. The relationship between gender and stockpiling was mediated by social learning (witnessing other people panic buying) and the perceived threat of COVID-19 (doomsday interpretations) while proximity and personal vulnerability to COVID-19 were non-significant. Results indicate that panic buying was influenced more by witnessing others stockpiling, personality, and catastrophic thinking rather than by proximity to danger. Education could target these factors in ongoing waves of the pandemic or future catastrophes.

## Introduction

Since the advent of the COVID-19 pandemic in 2020, there have been global news reports of widespread, excessive stockpiling and panic-buying of supplies such as toilet paper, food, hand sanitizer, and cleaning products. This panic-buying has led to supply shortages, empty shelves, and distress. It is unclear what psychological factors are associated with excessive stockpiling during the current COVID-19 pandemic. These behaviors have been described as panic-buying and hoarding which suggest links to anxiety. However, little is known about influences such as demographics, proximity or vulnerability to the virus, coping strategies, psychopathology, and personality.

The concept of “doomsday prepping” entered popular culture through a National Geographic Channel reality television series called Doomsday Preppers, which focussed on a subculture of people preparing for a post-apocalyptic world by hoarding supplies and weapons and rehearsing responses to hypothetical and often unlikely apocalyptic scenarios (Fetterman et al., 2019). Doomsday preppers in the pre-pandemic world often appeared to be extremists, performing elaborate drills in preparation for unlikely scenarios. There is little research on psychological characteristics associated with pre-COVID-19 survivalist prepping however an initial study found that it was associated with personality factors such as low agreeableness, paranoia, cynicism, conspiracy mentality, conservatism, and social dominance orientation (Fetterman et al., 2019).

The concept of the apocalypse appears in a variety of contexts throughout history and popular culture. Doomsday preppers are preoccupied in anticipating hypothetical scenarios that may bring about the end of civilization. The anticipated causes of doom differ markedly from person to person (Routledge et al., 2018). These survivalists or “preppers” typically secure places of shelter and stock up on food, water, medicine, fuel, and sometimes weapons. Many preppers are part of an online survivalist community. Despite any differences that may exist between preppers due to their personal visions of the apocalypse, they ultimately share the belief that there will be civil unrest and breakdown of law and order (Kabel and Chmidling, 2014).

The term doomsday prepping may evoke images of extremists, but it is also thought that prepping occurs on a continuum in the general population (Fetterman et al., 2019). On March 11, 2020, the World Health Organization (WHO) declared the novel Coronavirus (COVID-19) outbreak a global pandemic (World Health Organization, 2020). The excessive stockpiling during the pandemic suggests that psychological factors including doomsday interpretations may be important influences on these behaviors. While it is understandable to see an increase in purchases of essential supplies in readiness of the need to remain at home to avoid transmission, the extent of stockpiling has been excessive and has created inequitable situations that are risky to vulnerable people. There have been violent scenes as people fight over limited resources and some have left stores with nothing while others have excessive supplies.

Past pandemics have also fueled preparatory behaviors. Fung and Loke (2010) investigated the impact Severe Acute Respiratory Syndrome (SARS) had on Hong Kong families regarding preparatory behaviors. After the pandemic, most families considered infectious diseases their greatest worry and 68% of households had a food supply of 3 days, conveying that exposure to a global pandemic can influence preparatory behaviors. The spread of Influenza A (swine flu) also led to behavioral changes. One UK study reported that during the outbreak 20% of respondents had bought preparatory supplies (Goodwin et al., 2011). Swine flu's impact on daily living and societal functioning is dwarfed by that of COVID-19 and yet prepping behaviors emerged, albeit on a smaller scale.

While there is little research on stockpiling during COVID-19, one study found that consumers felt pressured to stockpile after seeing peers do the same (Zheng et al., 2020). Social learning theory (Bandura, 1977), states that behavioral conditioning and observational learning are mediated by cognitive processes such as motivation, therefore excessive preparatory responses may be learnt via social pressures which are then exacerbated by fear. Social influence and group interactions may be important factors in shaping behavior during crises (Abdulkareem et al., 2020) which require further examination in the context of stockpiling.

Community disease outbreaks can have profound impacts on mental health. A Hong Kong study on women discovered that during the SARS outbreak, women reported higher scores of depression and stress than they had prior (Yu et al., 2005). Increased levels of stress and depression were directly associated with feelings of fear, poor sleep, and financial loss. With its unpredictability, economic impact, and need for social isolation, COVID-19 has the potential to cause great mental strain as it demands the population to adapt in ways that are extremely novel to most (Horesh and Brown, 2020). It is thus important to investigate prepping in relation to coping and mental health symptoms.

There is limited research on those who identify as preppers and even less research on prepping in general populations. Recently, Fetterman et al. (2019) developed the Post-Apocalyptic and Doomsday Prepping Beliefs Scale (PAPBS) to assess prepping in the general population. The authors found that prepping beliefs were predictive of low agreeableness and humility, and positively correlated with cynicism, conspiracy mentality, and paranoia (Fetterman et al., 2019). Prepping beliefs were also related to conservatism and social dominance orientation (SDO), and the belief in the need to prep was associated with negative daily experiences and global political events. A limitation was that they did not include any measures of psychopathology, thus there is a need for further research.

There is ample evidence to suspect a potential link between prepping and anxiety, but no research has explored this yet. Those who engage in preparatory behaviors tend to express concerns surrounding resource availability and fears of others (Fetterman et al., 2019). This pessimistic outlook reflects both a distrust of humans and a fixation on negative future events, which can be an indicator of anxiety (Miranda and Mennin, 2004).

The anxious undertones of preparatory beliefs may stem beyond general anxiety to a more specific existential anxiety surrounding fears of uncertainty and death (Fetterman et al., 2019). Terror Management Theory (TMT) proposes that death-related cues can trigger personal and social defenses (Jonas et al., 2014). The juxtaposition of the inevitability of death with the desire for survival is a fundamental threat to the human self (Greenberg et al., 1997). In response to death cues and uncertainty, it is common to buffer existential anxiety through attempts to control one's environment and investment in groups (Jonas et al., 2014). Prepping may reflect an attempt to obtain control over a chaotic world and to ease anxiety related to mortality and potential chaos (Fetterman et al., 2019). Investing in groups is also evident in prepping culture. Many preppers engage in online forums which may strengthen their sense of security in their actions and even foster a sense of superiority as they tend to display great amounts of sociotropy, meaning they exhibit excessive investment in their social group. Morris and Johnson (2002) found that apocalyptic thinking was positively correlated with negative sociotropy, thus those high in apocalyptic thinking are likely to display hostility or disdain to those they consider “outsiders.”

Prepping may also bear similarities to obsessive-compulsive-like rituals. People with obsessive-compulsive disorder (OCD) will perform rituals to neutralize or reduce the distress they experience from a perceived threat (Jurgens et al., 2019). It is common amongst extreme preppers to rehearse the evacuation drills they intend to enact when their envisioned doomsday arrives (Kabel and Chmidling, 2014). Repeatedly rehearsing these drills in some ways resemble OCD-type rituals performed to prevent feared consequences (Jurgens et al., 2019). It may be that preparing for feared future eventualities temporarily eases anxiety, which then reinforces further prepping behavior when distressing cognitions arise. Additionally, thoughts of an impending apocalypse could show similarities to obsessions.

Hoarding excessive quantities of supplies that may never be used may resemble hoarding symptoms (Boerma et al., 2019). Approximately 20% of people with OCD experience hoarding symptoms, which are defined as the excessive accumulation of items regardless of their actual value (Matthews et al., 2014). There are differences between OCD-type symptoms and prepping behavior however, as OCD symptoms typically have an ego-dystonic quality, where the person feels compelled to perform them against their will. The extent to which this applies to doomsday prepping is unclear and there is a lack of research examining relationships between OCD-type symptoms and prepping.

Additionally, intolerance of uncertainty (IU) may be related to prepping beliefs and behaviors. IU refers to an aversive response to situations involving uncertainty where they are often perceived as threatening regardless of the true probability of threat (Tanovic et al., 2018). IU may be related to TMT as uncertainty around mortality can lead to an existential anxiety that causes one to seek control over their environment (Jonas et al., 2014). Therefore, prepping may relate to IU due to ongoing uncertainty in the pandemic.

IU is higher in those with OCD (Wheaton and Ward, 2020) and is also associated with subclinical obsessive-compulsive-type symptoms and need for control (Fourtounas and Thomas, 2016). Control may be particularly important as preppers display a large need to exert control over their environment (Fetterman et al., 2019).

It is not yet clear whether prepping behaviors in general and during the current pandemic differ by gender, however Fetterman et al. (2019) found that prepping beliefs are correlated with social dominance. Because males are often higher in SDO (Feather and McKee, 2012) and masculine socialization increases rates of SDO (Foels and Pappas, 2004), this may suggest a connection between prepping characteristics and traditionally masculine personality traits. In the context of the pandemic however, it is also possible that stockpiling may be linked to caring for dependent people such as children. Gender personality traits in relation to prepping have not yet been explored.

To summarize, while doomsday prepping has traditionally been considered an activity adopted by extremists, the COVID-19 pandemic has seen widespread preparatory behaviors in the general population. Prepping beliefs are influenced by negative daily experiences (Fetterman et al., 2019). The COVID-19 pandemic has resulted in significant changes to daily living such as loss of employment, working from home, financial impacts, restrictions on movement and social activities, all of which may contribute to stress (Horesh and Brown, 2020) and increase prepping beliefs (Fetterman et al., 2019). This study will examine preparatory beliefs and behaviors in the context of the COVID-19 pandemic in relation to demographic and psychological variables. There is anecdotal evidence to suggest that anxiety, obsessive-compulsive-like tendencies, IU, and traditional masculinity traits may be associated with prepping but there is no direct research. Results may inform the development of responses to crises by identifying which factors are associated with excessive stockpiling. Additionally, there is anecdotal information in the media about coping strategies being employed by people in the context of the unprecedented circumstances of the COVID-19 pandemic which require many people to shelter at home and physically isolate, however there is little research available about the nature and uptake of coping strategies. We also sought to ascertain the nature of coping strategies reported during the pandemic in order to better understand reactions to the pandemic.

The aims of the study are to:

1. Measure beliefs and behaviors related to doomsday prepping in a sample of people during the COVID-19 pandemic, as well as levels of psychopathology, coping strategies, and personality traits and whether these differ by gender.
2. Assess correlations between prepping beliefs and behaviors and other demographic and psychological variables.
3. Examine which constructs account for the most variance in prepping, and mediating factors.

## Methods

### Participants

The sample consisted of 384 participants (249 female), with ages ranging from 18 to 62 (*M* = 23.91, *SD* = 7.87). Participants were recruited via social media and through the university's research participation scheme for psychology students.

### Materials

#### Demographic Questionnaire

A demographic questionnaire was developed to collect information including age, country of birth and residence, gender, dependents, occupation, and level of education.

#### Prepping and Coping During a Pandemic Scale (PCP-Scale)

Due to the unprecedented nature of such a pandemic in recent history, there were no existing measures tailored to assess doomsday prepping, hoarding and stockpiling behaviors as well as other forms of coping in the context of a global health crisis. We therefore developed a measure to capture pandemic-specific aspects of doomsday prepping and coping, which were not assessed by existing doomsday prepping or general coping questionnaires (Supplementary Material). Due to the rapidly evolving crisis and the need to collect data while stockpiling was widespread during the pandemic, we did not generate items using some formal processes such as focus groups or the Delphi method. The items were generated based on expert and clinical input, discussion in university research symposia, qualitative analysis of media reports of common responses to the COVID-19 pandemic and themes related to stockpiling and coping and review of the small literature relating to stockpiling during previous pandemics (Fung and Loke, 2010; Goodwin et al., 2011). Similar approaches have been adopted in other COVID-19 research requiring rapid survey design (Ballou et al., 2020). Twenty reflective items of the questionnaire were developed which assessed preparatory behaviors (e.g., stockpiling), perceived motives for stockpiling (e.g., concerns about lockdowns), positive coping strategies (e.g., exercise, socializing online, focusing on personal goals) and beliefs about the pandemic (e.g., *I associate the current pandemic with a doomsday scenario*). We did not include questions about alcohol/drug use which were covered in existing coping questionnaires. Further contextual items such as proximity and vulnerability to COVID-19, prepping activity not related to COVID-19 and previous exposure to disasters were included to measure and control for COVID-19 threat level, general prepping tendencies and previous exposure to disasters, all of which may influence responses to the pandemic. Items were rated on a four point Likert scale with response corresponding to: 1 = *Not at all*, 2 = *Somewhat*, 3 = *Moderately*, 4 = *Very much so*. To gather more information in this new area of research, three open questions were included to ask respondents for any further information about their reasons for stockpiling, coping strategies and feared eventualities they were prepping for which were not included in the questionnaire. The scale produced a Cronbach's alpha of 0.72 indicating good internal consistency.

#### The Post-Apocalyptic and Doomsday Prepping Beliefs Scale (PAPBS)

The *PAPBS* is a 12-item questionnaire designed by Fetterman et al. (2019) to measure people's attitudes and beliefs about the post-apocalyptic world and prepping. Each item contains a statement that is rated on a 5-point Likert scale from *completely disagree* (1) to *completely agree* (5). The themes of *Humanity and resource concerns, Competitive survival*, and *Belief in the need to prep* serve as the three subscales for the questionnaire.

The *PAPBS* has been established to have good internal consistency yielding Cronbach's alphas ranging from 0.71 to 0.88 across studies (Fetterman et al., 2019).

#### The Survivalist Behavior Questionnaire (SBQ)

The *SBQ* (Jackson, 2018) focuses specifically on prepping behaviors that may be adopted by survivalists. The questionnaire contains eight items that explore prepping behaviors rated on a 5-point Likert scale ranging from strongly disagree (1) to strongly agree (5). Half of the items focus on physical behaviors (e.g., *I have stockpiled food and water to survive a potential major disaster*) and half assess planning behaviors (e.g., *I have a plan I could put into operation to survive a potential major disaster*). The overall scale has shown to have excellent internal reliability with a Cronbach's alpha of 0.90 and both subscales have been confirmed to have strong internal reliability both yielding a Cronbach's alpha of 0.87 (Jackson, 2018).

#### Generalized Anxiety Disorder Scale (GAD-7)

The *GAD-7* (Spitzer et al., 2006) is a brief measure developed to assess the extent of anxiety symptoms over the past fortnight. It is a seven item self-report measure that employs a 4-point Likert scale with responses ranging from 0 (*not at all*) to 3 (*nearly every day*). The scale has four cut off points of 0–4 (minimal anxiety), 5–9 (mild), 10–14 (moderate), and 15–21 (severe).

When tested amongst clinical populations the scale has shown to have excellent internal reliability with a Cronbach's alpha of 0.92 as well as good test-retest reliability with an intra class correlation of 0.83 (Spitzer et al., 2006). The questionnaire has demonstrated good construct, convergent, and factorial validity (Spitzer et al., 2006). The *GAD-7* is also a reliable measure of anxiety symptoms for general, non-clinical populations yielding a Cronbach's alpha of 0.89 (Lowe et al., 2008). Construct and factorial validity of the measure has also been supported in studies on non-clinical populations (Lowe et al., 2008).

#### The Obsessive-Compulsive Inventory- Revised (OCI-R)

The *OCI-R* (Foa et al., 2002) is a self-report measure of symptoms related to OCD. The questionnaire consists of 18 items that assess distress related to symptoms on a 5-point Likert scale ranging from 0 (*not at all*) to 4 (*extremely*). Individuals can score within a possible range of 0–72 with authors suggesting that a score of 21 or greater is indicative of likely OCD (Foa et al., 2002).

The *OCI-R* has shown to have good internal consistency across various clinical populations as well as non-clinical. The overall scale has yielded Cronbach's alpha coefficients ranging from 0.81 to 0.93 in populations with OCD, PTSD, Generalized Social Phobia (GSP), and non-anxious controls (Foa et al., 2002). The validity of the *OCI-R* is also evident in the significant positive correlations that have been observed between it and other measures of OCD (Foa et al., 2002).

#### The Depression Anxiety Stress Scale (DASS-21)

The *DASS-21* (Lovibond and Lovibond, 1995) explores distress experienced in the last 7 days. The measure contains 21 items that are scored on a 4-point Likert scale ranging from 0 (*did not apply to me at all*) to 3 (*applied to me very much, or most of the time*) with a total score between 0 and 63. The *DASS-21* has three subscales; *Depression, Anxiety*, and *Stress*.

The internal consistency for the three subscales is strong with Cronbach's alphas ranging from 0.87 to 0.94 and the scale also has good concurrent validity as it has moderate to strong correlations with other measures such as the Beck Depression Inventory, Beck Anxiety Inventory (Antony et al., 1998), and the Positive and Negative Affect Schedule (Henry and Crawford, 2005). The *DASS-21* has displayed excellent internal reliability within non-clinical populations with a Cronbach's alpha of 0.93 for the overall scale (Henry and Crawford, 2005).

#### The Intolerance of Uncertainty Scale- Short Form (IUS-12)

The *IUS-12* (Carleton et al., 2007) is a self-report measure that assesses a person's tendency to respond to uncertainty about the future negatively. The scale contains 12 items with a 5-point Likert response scale ranging from 1 (*not at all characteristic of me)* to 5 (*entirely characteristic of me*) to gauge how people cope with uncertain situations. The IUS-12 explores two factors, *Prospective IU* which refers to anxiety surrounding future events (7 items) and *Inhibitory IU* which refers to inhibited action or experience due to uncertainty (5 items).

The internal consistency of the *IUS-12* is excellent, the Cronbach's alpha of the overall scale is 0.91 and the two factors both yield alpha coefficients of 0.85 (Carleton et al., 2007). The internal consistency of the measure has been demonstrated across numerous clinical populations including those with OCD (Jacoby et al., 2013) as well as anxiety and depression (McEvoy and Mahoney, 2011). The *IUS-12* has also been established as a reliable measure of IU in non-clinical populations (Fourtounas and Thomas, 2016). The construct validity of the *IUS-12* has been demonstrated in several studies (Carleton et al., 2007; Jacoby et al., 2013).

#### The Brief Coping Orientation to Problems Experienced Scale (Brief COPE)

The *Brief COPE* (Carver, 1997) is a self-report measure of different coping strategies. The scale consists of 28 items that assess how people have utilized different coping styles to manage a hardship according to a 4-point Likert scale ranging from 1 (*I haven't been doing this at all*) to 4 (*I've done this a lot*). The *Brief COPE* measures two coping styles; *Avoidant Coping* and *Approach Coping*. Within these two composite subscales are 14 subscales (Cooper et al., 2008).

The *Brief COPE* has been established to have good internal consistency, the two subscales have both yielded Cronbach's alphas >0.72 (Cooper et al., 2008). Convergent and concurrent validity of the *Brief COPE* have also been confirmed via regression analyses (Cooper et al., 2008).

#### The Masculine Behavior Scale (MBS)

The *MBS* (Snell, 1989) is a self-report measure of gender-related behavioral tendencies. The instrument is constructed of 20 items that are scored using a 5-point Likert scale ranging from −2 (*disagree*) to +2 (*agree*). The *MBS* contains four subscales that explore traditionally masculine-related behaviors: *Success Dedication, Restrictive Emotionality, Inhibited Affection*, and *Exaggerated Self-reliance*. Each subscale can yield a total score of −10 to +10, more positive scores indicating an engagement in more stereotypically masculine behavior.

The *MBS* has demonstrated good internal consistency, the Cronbach's alpha of the subscales range from a low of 0.69 to a high of 0.89 and have reported test-retest coefficients at an average of 0.62 (Snell, 1989). The concurrent validity of the *MBS* has been confirmed through observed positive correlations with other measures of masculinity and femininity such as the Personal Attributes Questionnaire (PAQ; Spence and Helmreich, 1978).

### Procedure

The current study was reviewed and approved by the Institutional Human Research Ethics Committee. Participants were given information about the study and gave informed consent. Data were collected from 25th April to 28th August 2020.

### Statistical Analyses

Statistical Analyses of the data were conducted using the Statistical Package for the Social Sciences (SPSS) Version 26. Study variables were tested for violation of assumptions, missing data, and outliers. Responses with missing data were excluded from the analyses. After the deletion of incomplete responses, the final sample size was 378. A small number of outliers were detected in the sample, however results remained consistent after the removal of outliers and so the analyses reported include the outliers. The internal consistency of the psychometric instruments was assessed using Cronbach's alpha.

To explore the dimensionality of the new PCP-S questionnaire, an exploratory factor analysis with Varimax rotation was used to evaluate the factor structure of the PCP-S, with Eigen values >1.0 and visual inspection of the scree plot used to identify potential subscales. Cronbach's alpha was used to assess the internal consistency and also as a measure of reliability as it is equivalent to the mean of all split-half reliabilities (Warrens, 2015). Tests for normal distribution (skewness and kurtosis) were conducted for each item of the scale. Spearman's Rho was conducted between each PCP-S item and the Total score. Concurrent validity of the PCP-S was examined through correlations with other prepping, survivalist, coping and mental health measures which were hypothesized to be conceptually related to coping responses during the pandemic.

One-way Analyses of Variance (ANOVAs) were conducted to compare psychometric scores, prepping beliefs and behaviors, personality traits, and coping by gender. Pearson's correlational analyses were performed to evaluate the relationships between the measures of personality and psychopathology (anxiety, OCD-like symptoms, IU, and traditional masculinity traits) with prepping beliefs and behaviors. Linear regression analyses (LRAs) were performed to assess the extent to which each psychometric construct accounted for unique variance in prepping beliefs and behaviors beyond that explained by demographic variables. Finally, a parallel mediation analysis was conducted via PROCESS Macro Model 4, Version 3.5 (Hayes, 2018), using SPSS to examine which variables successfully mediate the relationship between gender and stockpiling.

## Results

### Demographic and Psychometric Characteristics of the Sample

Table 1 displays the demographic information and psychometric properties of the sample grouped by gender as well as the Cronbach's alpha coefficients of the scales. Of the sample, 250 participants were psychology students. The other participants worked in approximately 120 different occupations spanning health care, science, education, law, trades and engineering. Some common occupations were retail/hospitality (*n* = 60), and business owner (*n* = 15). Twenty were unemployed or furloughed, and one was a stay-at-home parent.

**Table 1:** Demographic and psychometric variables by gender (*N* = 373).

	**Overall** **(*n* = 384)**	**Male** **(*n* = 125)**	**Female** **(*n* = 248)**				
**Variable**	***M (SD)***	***M (SD)***	***M (SD)***	***F***	***p***	**Partial **η**^2^**	***a***
Age (Years)	23.87 (7.79)	26.26 (9.01)	22.54 (6.78)	18.28	<0.001	0.04	
Dependents	0.51 (1.05)	0.56 (1.08)	0.49 (1.06)	0.34	0.563	0.01	
Years of education	14.77 (2.55)	14.97 (2.73)	14.68 (2.48)	2.00	0.159	0.01	
PAPBS	31.85 (7.38)	34.26 (8.66)	30.68 (6.33)	16.64	<0.001	0.04	0.81
SBQ	13.18 (5.56)	15.52 (6.07)	12.04 (4.92)	30.64	<0.001	0.08	0.84
GAD-7	7.47 (5.43)	7.19 (5.53)	7.65 (5.37)	0.57	0.452	0.01	0.91
OCI-R	17.06 (12.47)	17.68 (11.96)	16.81 (12.72)	0.41	0.525	0.00	0.90
DASS-21	17.24 (11.86)	16.82 (11.89)	17.48 (11.87)	0.25	0.620	0.01	0.93
IUS-12	30.61 (9.28)	31.04 (9.07)	30.43 (9.40)	0.36	0.549	0.00	0.89
COPE Avoidant	25.50 (5.76)	25.45 (6.17)	25.54 (5.57)	0.01	0.904	0.00	0.76
COPE Approach	31.33 (6.83)	30.14 (6.51)	32.84 (6.89)	5.00	0.026	0.01	0.86
MBS	4.30 (14.16)	7.78 (15.41)	2.66 (13.24)	9.37	0.003	0.02	0.89

PAPBS, Post-Apocalyptic and Doomsday Prepping Beliefs Scale; SBQ, Survivalist Behavior Questionnaire; GAD-7, Generalized Anxiety Disorder-7; OCI-R, Obsessive-Compulsive Inventory-Revised; DASS-21, Depression Anxiety Stress Scale-21; IUS-12, Intolerance of Uncertainty Scale- Short Form; MBS, Masculine Behavior Scale.

*Participants who did not answer the gender question (n = 5) were excluded from the gender comparative analyses*.

Most participants resided in Australia (*n* = 259), with the remainder spanning 27 other countries [Andorra (*n* = 1), Armenia (*n* = 1), Austria (*n* = 2), Belgium (*n* = 1) Brazil (*n* = 1), Bulgaria (*n* = 1), Canada (*n* = 6), Czech Republic (*n* = 1), Finland (*n* = 1), France (*n* = 2), Germany (*n* = 7), Hong Kong (*n* = 1), India (*n* = 3), Ireland (*n* = 1), Italy (*n* = 1), Japan (*n* = 1), Liechtenstein (*n* = 1), Malaysia (*n* = 1), Mexico (*n* = 2), Netherlands (*n* = 16), New Zealand (*n* = 1), Norway (*n* = 4), Portugal (*n* = 1), Spain (*n* = 3), United Arab Emirates (*n* = 1), United Kingdom (*n* = 13), USA (*n* = 32)]. Prior to analyses, normality was assessed via skewness and kurtosis statistics, which indicated that the sample met normality assumptions except for items 18, 19 and 20 of the PCP-S (seeking help from a doctor, psychologist or help-line) which showed floor effects. The homogeneity of variance between the male and female groups was violated and so the Welch ANOVA statistic is reported. Males were significantly older than females, but males and females did not differ significantly in years of education or number of dependents. Males averaged higher scores on the *PAPBS* and the *SBQ* than females. There were no significant differences between genders on the other psychological and personality measures of the study except for masculinity (*MBS*) where, as expected, males averaged higher scores, and approach-focused coping (*COPE*) where females averaged higher scores than males.

### Properties of the New Prepping and Coping During a Pandemic Scale (PCP-S)

Table 2 displays the descriptive information for the items of the PCP-S for the current sample, by gender. The most common reported reasons for stockpiling were concerns about lockdowns, responding to others stockpiling, and fears of getting sick. Males reported higher rates of stockpiling, and higher rates of stockpiling in response to observing others panic buying (social learning) than females. Males and females did not differ significantly in their proximity or vulnerability to COVID-19 nor did they differ significantly in their concern about society's future due to COVID-19. However, males were significantly more likely to associate the current pandemic with a doomsday scenario. The most common reported strategies for remaining positive during COVID-19 were socializing via technology, focusing on personal goals or hobbies, and looking for positives that emerged during the pandemic such as reductions in pollution. Females reported significantly more socializing via technology, spending time with pets, and consulting a doctor than males. Males reported focusing on personal interests significantly more than females.

**Table 2:** Prepping and Coping during a Pandemic Scale (PCP-Scale), Mean Scores and Response Percentages (*N* = 373).

	**Male (*n* = 125)**	**Female (*n* = 248)**				**Overall Sample**			
						**Response percentages**			
Variable	*M (SD)*	*M (SD)*	*F*	*P*	partial η^2^	1	2	3	4
Proximity to COVID-19	2.40 (0.83)	2.41 (0.92)	0.01	0.945	0.00	10.7%	51.7%	25.7%	8.5%
Vulnerability to COVID-19	1.42 (0.80)	1.47 (0.82)	0.27	0.606	0.00	71%	17.4%	7.5%	4.2%
Stockpiling	2.08 (1.10)	1.63 (0.68)	17.61	<0.001	0.09	44.2%	40.4%	8.5%	6.9%
**Stockpiling Reasons**									
Fears of supermarkets closing	1.63 (0.88)	1.56 (0.78)	0.57	0.452	0.02	54.9%	24.6%	10.2%	2.9%
Fears of lockdown	2.06 (0.97)	1.85 (0.94)	3.58	0.060	0.01	39.4%	28.4%	17.6%	6.9%
In response to others	2.14 (1.24)	1.87 (1.01)	4.11	0.044	0.02	44.5%	20.9%	15.4%	12.8%
Friend and family advice	1.48 (0.89)	1.52 (0.77)	0.12	0.737	0.00	61.1%	20.1%	7.5%	3.7%
Fears of getting sick	1.89 (1.06)	1.79 (0.93)	0.74	0.392	0.03	45.5%	24.1%	15%	7.7%
Associating COVID-19 with doomsday	1.99 (0.91)	1.81 (0.76)	3.53	0.047	0.02	36.4%	45.5%	14.2%	4.5%
Concern about society's future	2.67 (1.01)	2.76 (0.84)	0.65	0.421	0.01	7.2%	37.3%	31.9%	23.8%
**Coping Strategy**									
Socializing via technology	2.70 (0.88)	3.02 (0.82)	11.45	0.001	0.03	5.1%	26%	41.8%	27.3%
Support forums	1.51 (0.86)	1.64 (0.83)	2.00	0.158	0.01	60%	24.1%	12.6%	3.4%
Personal interests	3.11 (0.94)	2.87 (0.90)	5.88	0.016	0.02	6.7%	25.5%	35.1%	32.9%
Exercise	2.47 (1.07)	2.45 (1.02)	0.05	0.818	0.00	20.1%	35.6%	23.6%	20.9%
Practicing meditation	1.65 (0.91)	1.80 (0.93)	2.21	0.139	0.01	51.5%	30%	11.5%	7.2%
Spiritual practice	1.58 (0.94)	1.60 (0.93)	0.02	0.882	0.00	65.7%	16%	12.3%	10.4%
Home improvements	2.10 (0.95)	2.28 (0.96)	3.23	0.074	0.01	26.8%	35.1%	27.9%	10.4%
Spending time with pets	2.14 (1.22)	2.43 (1.23)	4.42	0.037	0.01	38.9%	15.5%	20.4%	25.5%
Positive impacts of COVID-19	2.45 (1.02)	2.66 (0.97)	3.57	0.060	0.01	16.3%	29.5%	33.8%	20.6%
Consulting a doctor	1.22 (0.64)	1.37 (0.68)	4.16	0.042	0.02	77.5%	14.7%	6.7%	1.3%
Consulting a psychologist	1.22 (0.69)	1.37 (0.83)	3.33	0.069	0.01	83.1%	6.9%	5.4%	3.8%
Phoning a helpline	1.06 (0.32)	1.11 (0.43)	1.80	0.180	0.01	94.3%	3.2%	2.1%	0.54%

*Scores correspond to the following qualitative descriptors of the response scale: 1 = Not at all, 2 = Somewhat, 3 = Moderately, 4 = Very much so. Participants who did not answer the gender question (n = 5) were excluded from the gender comparative analyses*.

An exploratory factor analysis was conducted on reflective questions, items 1–20, but not including the contextual (control) or open questions, to explore the dimensionality of the questionnaire. The Kaise-Meyer-Olkin index of sampling adequacy was 0.74 and Bartlett's sphericity test was significant (χ2 = 1,654.24, *p* < 0.001), indicating that the PCP scale data from our survey were suitable for factor analysis. No item detracted from the scale alpha.

Principal components analysis revealed six factors with eigenvalues >1, accounting for 62.23% of the variance (Factor 1: 22.34%; Factor 2: 12.46%; Factor 3: 8.31%; Factor 4: 7.38%; Factor 5: 5.93%; Factor 6: 5.78%). All 20 reflective items of the PCP-S loaded robustly onto one of the identified factors rotated with Varimax method, with no overlap when loadings of <0.36 were eliminated (Table 3). The anti-image correlation matrix indicated that all measures of sampling adequacy were above the acceptable level of 0.50.

**Table 3:** Component loading values of the six-factor structure of the Prepping and Coping during a Pandemic Scale.

	**Factor**					
	**1**	**2**	**3**	**4**	**5**	**6**
1. Overall stockpiling during COVID-19	0.711					
2. Stockpiling due to fears/concerns about supermarkets closing	0.731					
3. Stockpiling due to fears/concerns about restrictions on going out, or lockdowns	0.818					
4. Stockpiling in response to other people buying excessive amounts of supplies	0.637					
5. Stockpiling because friends or family advised to	0.639					
6. Stockpiling due to fears about getting sick and not being able to go out	0.762					
7. Associating COVID-19 with a “doomsday” scenario				0.781		
8. Worrying about the future of society because of COVID-19				0.750		
9. Socializing with friends and family via technology			0.579			
10. Reading or engaging in support forums		0.502				
11. Focusing on personal interests/goals/hobbies			0.656			
12. Keeping positive through exercise			0.624			
13. Practicing meditation/mindfulness					0.783	
14. Engaging in spiritual practice or thought					0.808	
15. Home maintenance/improvements			0.523			
16. Spending time with pets.						0.787
17. Looking for positives, e.g., reductions in pollution, the return of animals to certain areas, etc.						0.680
18. Consulting a doctor		0.750				
19. Consulting a psychologist		0.772				
20. Phoning a helpline		0.662				

*Extraction Method: Principal Component Analysis. Rotation method: Varimax with Kaiser normalization. Factors correspond to themes of: 1“Stockpiling,” 2 “Formal help,” 3 “Self-care/hobbies,” 4 “Doomsday cognitions,” 5 “Mindfulness/spirituality,” 6 “Nature and animals”*.

As shown in Table 4, each item was significantly correlated with the Total score, with small to large correlations (0.17–0.64). Additionally, correlations between each of the six factors and the Total score were significant and moderate to large (0.38-0.74). The positive correlations between each item and each subscale with the Total score suggest that the questionnaire is measuring a range of related cognitive and behavioral responses to the pandemic, as well as providing a specific measure of pandemic-related stockpiling. The PCP-S Total showed low-moderate significant correlations with traditional doomsday prepping and survivalist questionnaires, psychopathology measures (anxiety and OCD-related) and stress (Table 5), which were theoretically related concepts, supporting concurrent validity of the scale. The positive correlations between the PCP-S Total, stress and psychopathology (Table 5) also suggest that while some coping methods (e.g., meditation, exercising) may be considered more “positive,” and others (e.g., stockpiling) might be considered undesirable, all these coping methods are positively related to the stressful nature of the pandemic. The Total score may therefore potentially be used as a measure of overall cognitive and behavioral responses to the pandemic, in addition to the prepping scores.

**Table 4:** Spearman Rho's correlations between each PCP-S item, factors and the total score.

**Item**	***r***
1. Overall stockpiling during COVID-19	0.583^**^
2. Stockpiling due to fears/concerns about supermarkets closing	0.476^**^
3. Stockpiling due to fears/ concerns about restrictions on going out, or lockdowns	0.641^**^
4. Stockpiling in response to other people buying excessive amounts of supplies	0.545^**^
5. Stockpiling because friends or family advised to	0.547^**^
6. Stockpiling due to fears about getting sick and not being able to go out	0.529^**^
7. Associating COVID-19 with a “doomsday” scenario	0.379^**^
8. Worrying about the future of society because of COVID-19	0.339^**^
9. Socializing with friends and family via technology	0.274^**^
10. Reading or engaging in support forums	0.408^**^
11. Focusing on personal interests/goals/hobbies	0.358^**^
12. Keeping positive through exercise	0.370^**^
13. Practicing meditation/mindfulness	0.368^**^
14. Engaging in spiritual practice or thought	0.349^**^
15. Home maintenance/improvements	0.367^**^
16. Spending time with pets	0.288^**^
17. Looking for positives, e.g., reductions in pollution, the return of animals to certain areas, etc.	0.326^**^
18. Consulting a doctor	0.170^**^
19. Consulting a psychologist	0.181^**^
20. Phoning a helpline	0.312^**^
**Factors**	
1. *Stockpiling*	0.742^**^
2. *Formal help*	0.379^**^
3. *Self-care/hobbies*	0.542^**^
4. *Doomsday cognitions*	0.412^**^
5. *Mindfulness/spirituality*	0.434^**^
6. *Nature/animals*	0.381^**^

PCP, Prepping and Coping during a Pandemic Scale.

** *Correlation is significant at p < 0.01*.

**Table 5:** Correlations for Study Variables (*N* = 373).

**Variable**	**1**	**2**	**3**	**4**	**5**	**6**	**7**	**8**	**9**	**10**	**11**	**12**
1. PAPBS	–											
2. SBQ	0.55^**^	–										
3. GAD-7	0.24^**^	0.15^**^	–									
4. OCI-R	0.29^**^	0.19^**^	0.46^**^	–								
5. DASS-21 Overall	0.22^**^	0.12^*^	0.78^**^	0.50^**^	–							
6. DASS Depression	0.15^**^	0.06	0.59^**^	0.34^**^	0.86^**^	–						
7. DASS Anxiety	0.11^*^	0.02	0.65^**^	0.49^**^	0.84^**^	0.61^**^	–					
8. DASS Stress	0.29^**^	0.21^**^	0.76^**^	0.46^**^	0.87^**^	0.59^**^	0.63^**^	–				
9. IUS-12	0.34^**^	0.19^**^	0.55^**^	0.45^**^	0.58^**^	0.43^**^	0.50^**^	0.57^**^	–			
10. IUS Prospective	0.37^**^	0.26^**^	0.49^**^	0.36^**^	0.48^**^	0.33^**^	0.36^**^	0.54^**^	0.91^**^	–		
11. IUS Inhibitory	0.22^**^	0.06	0.48^**^	0.43^**^	0.54^**^	0.43^**^	0.53^**^	0.45^**^	0.85^**^	0.54^**^	–	
12. MBS	0.26^**^	0.18^**^	0.14^**^	0.18^**^	0.17^**^	0.12^*^	0.07	0.23^**^	0.29^**^	0.31^**^	0.17^**^	–
13. PCP-S Total	0.348^**^	0.366^**^	0.205^**^	0.322^**^	0.102	−0.028	0.112^*^	0.182^**^	0.117^*^	0.178^**^	0.008	0.021

PAPBS, Post-Apocalyptic and Doomsday Prepping Beliefs Scale; SBQ, Survivalist Behavior Questionnaire; GAD-7, Generalized Anxiety Disorder-7; OCI-R, Obsessive-Compulsive Inventory-Revised; DASS-21, Depression Anxiety Stress Scale-21; IUS-12, Intolerance of Uncertainty Scale- Short Form; MBS, Masculine Behavior Scale, PCP-S, Prepping and Coping during a Pandemic Scale.

* p < 0.05,

** *p < 0.01*.

Responses to the open question about additional reasons for stockpiling resources during COVID-19 included fear of going out and catching COVID-19, financial worries (economic downturn or collapse, job loss, price increases and not being able to afford resources later on), fear of the collapse of civilization due to multiple threats (climate change, natural disasters, fires, earthquakes, drought, flood, terrorism, or solar flare), fear of the grid going down, fear of collapse of supply chains (stores running out of some items like meat/produce, no bread to feed the children, worried that essentials like feminine hygiene products would run out), stocking up to be ready to bug out to a remote, off-grid location, stockpiling due to previous experience of war, and wanting to help neighbors.

Responses to the open questions about any other coping strategies used included electronic entertainment (watching more television series, streaming, radio, podcasts, gaming, listening to music, watching pornography), food (cooking, eating more healthily), spending more time with loved ones (family, partners, spending precious time with children) focusing on self-improvement (studying, reading books, journaling), thinking positively (only looking at positive news stories and vaccine development, pretending it's not happening), finding out more about the virus (watching the world cases, researching literature on the virus), sleeping, oversleeping, masturbation, using weed/alcohol, family cocktail nights at home and partying with roommates.

Answers to the open question about whether people were preparing for anything other feared events not listed included mass civil disobedience, societal collapse, loss of jobs due to automation, and a post-brexit slump.

### Correlational Analyses

Two-tailed Pearson's correlational analyses indicated that prepping beliefs measured by the *PAPBS* were positively associated with the *GAD-7, OCI-R, DASS-21, IUS-12*, and *MBS* (Table 5). Additionally, prepping behaviors as measured by the *SBQ* positively correlated with the *GAD-7, OCI-R, DASS-21, IUS-12*, and *MBS*. Unlike prepping beliefs, prepping behaviors were only significantly related to the *Stress* subscale of the *DASS-21* and the *Prospective IU* subscale of the *IUS-12*. The PCP-S Total score correlated significantly with general prepping measures (PAPBS and SBQ), psychopathology (GAD-7, OCI-R, DASS Anxiety), Stress and IU.

### Linear Regression Analyses (LRAs)

Three linear regressions were conducted to assess the extent to which each psychometric construct accounted for unique variance in prepping characteristics (as measured by the *PAPBS, SBQ*, and self-reported stockpiling due to COVID-19) while controlling for age and gender.

The presence of univariate outliers was revealed through boxplots but as the pattern of results remained consistent regardless of their removal the outliers were retained for the analysis. The presence of multivariate outliers was indicated as Mahalanobis distance exceeded the critical *X*^2^ for *df* = 7 (at α = 0.001) of 24.32 for two cases. Likewise, as the pattern of results after the multivariate outliers' removal remained consistent with the results prior to their removal, the multivariate outliers were retained.

The first LRA investigated the variance explained by psychometric constructs on prepping beliefs as measured by the *PAPBS* while controlling for age and gender. At step 1, age and gender as well as the *GAD-7, OCI-R, DASS-21, IUS-12*, and *MBS* were entered in the regression model, the combined demographic and psychometric variables accounted for 52% of the variance in prepping beliefs, *R*^2^ = 0.27, *F*_(7,342)_ = 17.85, *p* < 0.001. The values of unstandardized (B) and standardized (β) regression coefficients, and semi-partial correlations (*sr*^2^) for each predictor in the regression model are reported in Table 6. Significant predictors were age, gender, the *OCI-R, IUS-12*, with the *MBS*. Age (β = 0.25, *p* < 0.001) and IU (β = 0.21, *p* = 0.001) being the strongest predictors.

**Table 6:** Linear Regression Predicting Prepping Beliefs as measured by the PAPBS (*n* = 373).

**Predictor**	**B**	**β**	**sr^2^**	***p***
Age	0.23	0.25^**^	0.27	<0.001
Gender	−2.10	−0.14^*^	−0.15	0.006
GAD-7	0.14	0.10	0.07	0.187
OCI-R	0.11	0.19^*^	0.18	0.001
DASS-21	−0.06	−0.10	−0.07	0.198
IUS-12	0.16	0.21^*^	0.18	0.001
MBS	0.09	0.18^**^	0.19	<0.001

PAPBS, Post-Apocalyptic and Doomsday Prepping Beliefs Scale; GAD-7, Generalized Anxiety Disorder-7; OCI-R, Obsessive-Compulsive Inventory-Revised; DASS-21, Depression Anxiety Stress Scale-21; IUS-12, Intolerance of Uncertainty Scale- Short Form; MBS, Masculine Behavior Scale.

* p < 0.01,

** *p < 0.001*.

The second LRA examined the relative utility of the psychometric constructs in predicting variance in prepping behaviors as measured by the *SBQ* while controlling for age and gender. At step 1, age and gender, the *GAD-7, OCI-R, DASS-21, IUS-12*, and *MBS* were entered and the psychometric constructs combined with age and gender accounted for 49% of variance in prepping behaviors, *R*^2^ = 0.24, *F*_(7,342)_ = 15.02, *p* < 0.001. Significant predictors for prepping behaviors were age, gender, the *OCI-R*, and the *MBS* (Table 7).

**Table 7:** Linear Regression Predicting Prepping Behaviors as measured by the SBQ (*n* = 373).

***Predictor***	***B***	**β**	**sr^2^**	***p***
Age	0.22	0.30^**^	0.31	>0.001
Gender	−2.51	−0.21^**^	−0.20	>0.001
GAD-7	0.14	0.14	0.09	0.064
OCI-R	0.08	0.17^*^	0.17	0.002
DASS-21	−0.06	−0.12	−0.08	0.136
IUS-12	0.04	0.07	0.06	0.251
MBS	0.04	0.11^*^	0.12	0.025

SBQ, Survivalist Behavior Questionnaire; GAD-7, Generalized Anxiety Disorder-7; OCI-R, Obsessive-Compulsive Inventory-Revised; DASS-21, Depression Anxiety Stress Scale-21; IUS-12, Intolerance of Uncertainty Scale- Short Form; MBS, Masculine Behavior Scale.

* p < 0.05,

** p < 0.001.

The final LRA explored the variance explained by psychometric constructs on stockpiling in response to COVID-19 (item 3 on the PCP-S) while controlling for age and gender. At step 1, age and gender, the *GAD-7, OCI-R, DASS-21, IUS-12*, and *MBS* were entered in the regression model. Demographic and psychometric variables combined accounted for 51% of variance in COVID-19 stockpiling, *R*^2^ = 0.26, *F*_(7,342)_ = 17.11, *p* < 0.001. Age, gender, the *GAD-7, OCI-R*, and the *MBS* were the strongest predictors of stockpiling (Table 8).

**Table 8:** Linear Regression Predicting Stockpiling as measured be the Prepping and Coping during a Pandemic Scale (PCP-S) (*n* = 373).

**Predictor**	**B**	**β**	**sr^2^**	***p***
Age	0.04	0.39^**^	0.40	<0.001
Gender	−0.23	−0.13^*^	−0.14	0.010
GAD-7	0.03	0.21^*^	0.15	0.007
OCI-R	0.01	0.12^*^	0.11	0.038
DASS-21	−0.01	−0.08	−0.06	0.290
IUS-12	0.01	0.03	0.02	0.688
MBS	0.01	0.13^*^	0.14	0.012

GAD-7, Generalized Anxiety Disorder-7; OCI-R, Obsessive-Compulsive Inventory-Revised; DASS-21, Depression Anxiety Stress Scale-21; IUS-12, Intolerance of Uncertainty Scale- Short Form; MBS, Masculine Behavior Scale.

* p < 0.05,

** *p < 0.001*.

### Mediation Analyses

**Figure 1:**
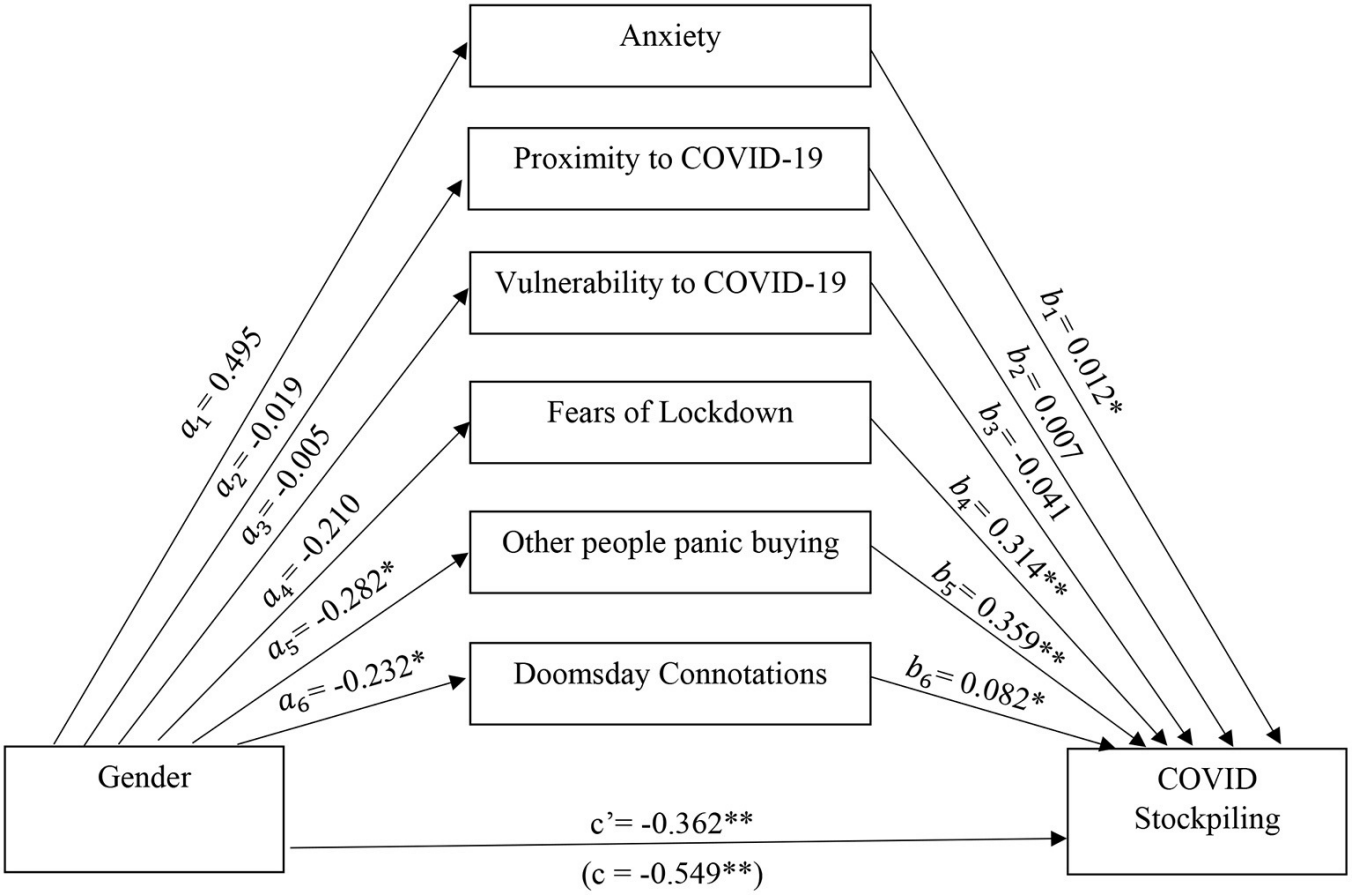
Mediating effects of perceived and actual threat of COVID-19 and social learning on stockpiling (*n* = 373). *p < 0.05, **p < 0.001; *a*_*n*_ = effect of gender on variables; *b*_*n*_ = effect of variables on COVID stockpiling; c′= direct effect of gender on stockpiling; c = total effect of gender on stockpiling.

To further examine the relationship between gender and stockpiling, a parallel mediation analysis (displayed in Figure 1) was conducted using PROCESS Macro Model 4 in SPSS. The analysis examined which variables significantly mediated the relationship between gender and stockpiling. The mediators included potential vulnerability to contracting COVID-19 (such as proximity to active cases or personal health vulnerability), doomsday-like perceptions of COVID-19, concerns about lockdowns, and responding to other people's excessive panic buying. As anxiety was a significant predictor of stockpiling it was also included as a mediator to see if it explained the relationship between gender and stockpiling.

Anxiety was not a significant mediator (*b* = 0.006(0.009), 95% CI [−0.008, 0.029]) suggesting that while anxiety had a significant effect on stockpiling it does not account for higher rates of stockpiling in males. Witnessing other people buying excessive amounts of supplies significantly mediated the relationship between gender and stockpiling (*b* = −0.101(0.052), 95% CI [−0.213, −0.008]). Fears of lockdown was not a significant mediator (*b* = 0.011(0.007), 95% CI [−0.056, 0.078]), suggesting that males stockpiling at a higher rate is related to social learning. Additionally, associating the current pandemic with a doomsday scenario significantly mediated the relationship between gender and stockpiling (*b* = −0.019 (0.018), 95% CI [−0.057, −0.008]), suggesting that males interpreting COVID-19 as a doomsday scenario was related to their greater stockpiling. However, proximity to COVID-19 cases and personal vulnerability to COVID-19 were not significant mediators, indicating that doomsday connotations or catastrophic thinking in relation to COVID-19, along with social learning were more powerful than proximity or personal vulnerability to COVID-19 in influencing stockpiling.

### Summary

First, one-way ANOVAs confirmed that males scored higher on the *PAPBS* and *SBQ* and also reported higher rates of stockpiling in response to COVID-19, although males and females did not differ significantly in their proximity and vulnerability to COVID-19 nor did they differ significantly in psychological symptoms. The new PCP-S scale showed adequate reliability and concurrent validity. Correlational analyses revealed significant positive associations between prepping beliefs and behaviors and anxiety, OCD-like symptoms, IU, and traditional masculinity traits. LRAs revealed that age and gender were consistent significant predictors across all measures of prepping. Additionally, LRAs indicated that OCD-like symptoms, IU, and traditional masculinity accounted for the most unique variance in predicting prepping beliefs (measured by the *PAPBS*). In comparison, traditional masculinity and OCD-like symptoms were significant predictors for prepping behaviors (measured by the *SBQ*) and, with the addition of anxiety, were significant predictors for COVID-19 stockpiling (PCP-S). A parallel mediation analysis indicated two psychological variables (doomsday connotations and seeing other people panic buying) mediated the relationship between gender and stockpiling while variables related to the actual danger of COVID-19 such as proximity or personal vulnerability were non-significant.

## Discussion

This study investigated doomsday prepping during the COVID-19 pandemic to better understand global waves of resource hoarding and panic buying. We aimed to investigate the nature of preparatory beliefs and behaviors and the associated sociodemographic, psychopathology, and personality constructs. This is an area of key importance in the context of the current pandemic as there is limited research on what drives excessive stockpiling behaviors which have led to distress, conflict, and negative ramifications on daily living.

The overall sample reported fears of lockdown, seeing others stockpile, and fears of getting sick as their most common reasons for stockpiling due to COVID-19, showing the importance of feared future outcomes to hoarding and panic-buying. Males reported stockpiling due to COVID-19 at significantly higher rates than females and had higher scores on measures of doomsday prepping beliefs and behaviors. Similarly, traditional masculinity traits correlated positively with all prepping measures. These are novel findings in the literature which are somewhat consistent with previous findings of links between prepping beliefs, social dominance orientation, and competitiveness (Fetterman et al., 2019) which have both been observed to occur more in males (Apicella et al., 2011; Feather and McKee, 2012).

We assessed self-reported levels and reasons for stockpiling during the pandemic, using a scale developed for the current study. Males reported being influenced to stockpile when observing others panic-buying at higher rates than females, suggesting social learning processes. Additionally, males were more likely to interpret the current pandemic as a doomsday scenario than were females. Additionally, there were no gender differences in the measures of psychopathology (anxiety, OCD-type symptoms, and IU). This suggests that higher prepping characteristics in males were related to personality traits seen in traditional masculinity, as well as social learning and catastrophic thinking. The findings of the link between traditional masculinity traits and prepping are perhaps not surprising, as traditional masculinity traits include being in control, in charge and not relying on others, all of which are conceptually related to self-sufficiency. Traditional male traits may fuel competitiveness and a perceived need to compete for supplies and display an exaggerated self-reliance when confronted with images of others hoarding resources (Zheng et al., 2020).

Prepping characteristics were positively related to anxiety, obsessive-compulsive-like symptoms, and intolerance of uncertainty. The relationship between prepping characteristics and anxiety may be explained by negative future-based thinking that is seen in anxious individuals (Miranda and Mennin, 2004) which can be compared to the pessimistic outlook that serves as a foundation for prepping (Fetterman et al., 2019). In the context of the current pandemic, many people have lost their source of employment, social connections, and receive frequent reminders via the media of the negative ramifications of COVID-19 such as shortages, social unrest, and economic fallout (Horesh and Brown, 2020). In turn, this negative outlook may fuel beliefs in the need to prep (Fetterman et al., 2019).

It was unclear how much prepping and obsessive-compulsive-like symptoms would relate, due to a lack of previous research. The OCD-symptom measure used in the current study examines symptoms that occur on a continuum related to alleviating intrusive thoughts about feared outcomes (Foa et al., 2002). Its positive correlation with prepping measures suggests a potential overlap between intrusive thoughts, which may elevate beliefs in the need to prep. Therefore, people may be attempting to soothe distress associated with these intrusive thoughts via prepping behaviors.

The association between prepping characteristics and intolerance of uncertainty is consistent with research that suggests IU is related to fears in one's ability to cope with unpredictability (Jensen et al., 2016), which may manifest in self-reassuring behaviors like stockpiling. Furthermore, the positive association between anxiety and IU and prepping characteristics supports the propositions of TMT (Greenberg et al., 1997). As individuals feel uncertain about the future and receive a heightened number of death cues due to COVID-19, they may attempt to ease their existential anxiety through prepping behaviors such as stockpiling as it allows them to seek control over a chaotic environment (Jonas et al., 2014).

The *Stress* subscale of the *DASS-21* had the strongest correlation with prepping behaviors (measured by the *SBQ*) while the *Anxiety* and *Depression* subscales were non-significant. Stress, by definition is an appraisal of not having sufficient resources to cope with the demands of a situation, and therefore may be a catalyst for preparatory behaviors during the current pandemic. Research during the 2003 SARS outbreak demonstrated how the crisis led to elevated levels of stress (Yu et al., 2005), however we are not aware of previous research linking stress levels to stockpiling.

Next, we examined which psychological constructs predicted prepping. The variables were not all significant predictors across the different prepping measures. OCD-type symptoms and traditional masculinity were significant predictors of prepping beliefs, behaviors, and COVID-19 stockpiling, while IU was only predictive of prepping beliefs, and anxiety was only predictive of COVID-19 stockpiling.

OCD-like symptoms were a significant predictor of all prepping measures. Prepping may be interpreted as a coping mechanism to alleviate intrusive thoughts related to a post-apocalyptic world (Jurgens et al., 2019). Preparing for feared future outcomes may temporarily ease anxiety, which then reinforces prepping behaviors like stockpiling when anxious cognitions arise. Traditional masculinity was also a significant predictor of all measures indicating that features of stereotypical hyper-masculinity like an exaggerated self-reliance may promote the evolution of prepping characteristics. Traditional masculinity remained a significant predictor when gender was controlled for which further supports the argument that it is not merely males engaging in prepping actions but those who possess a greater degree of these stereotypical masculine traits like competitiveness and dominance.

Age was a significant predictor of all prepping measures. This is a novel finding for the field of research. Males in this sample not only reported higher prepping scores but were also significantly older than females. This raises the possibility that it is not traits of traditional masculinity alone accounting for males' greater prepping scores but perhaps also age, however multivariate regression analyses indicated that both male gender and age significantly accounted for unique variance in prepping scores.

Finally, this study sought to understand which factors mediated the relationship between gender and stockpiling during the pandemic. Social learning (witnessing panic-buying) and catastrophic thinking (doomsday interpretations of the pandemic) were found to significantly mediate the relationship between gender and stockpiling, however objective threat of COVID-19 (proximity and vulnerability) did not. This suggests that males' greater stockpiling is associated with constructs other than rational risk appraisal, including catastrophic thinking related to COVID-19 and a sense of competition with others for supplies. This novel finding may explain why stockpiling has persisted even when risks reduce, and reassurances are given by authorities and suppliers.

Observing others panic buying was a significant mediator for the relationship between gender and stockpiling indicating that social learning has a substantial impact on preparatory responses during COVID-19. This finding supports the research of Zheng et al. (2020) which found that consumers panic buying during a crisis is heavily influenced by social learning. Stockpiling may manifest through observational learning which is mediated through motivations. For example, the motivation to suppress feelings of fear that are produced through catastrophic thinking (Bandura, 1977). This finding signifies the importance of group interactions in shaping responses to a crisis, which is consistent with the research of Abdulkareem et al. (2020) on how collective learning is instrumental in behavioral change in an epidemic. These findings convey that prepping beliefs and behaviors may be fuelled by psychological factors other than objective threat and that social learning and catastrophic cognitions may be the root of some prepping behaviors.

Additionally we examined a range of coping strategies during the unfolding health crisis. The most common reported strategies were socializing via technology, focusing on personal goals or hobbies, and looking for positives that emerged during the pandemic such as reductions in pollution. Females socialized more via technology, spent more time with pets, and more frequently consulted a doctor than males. Males reported significantly more focusing on personal interests than females. Uptake of formal help, such as through health care professionals, was lower than other strategies, consistent with many pre-pandemic studies (Thomas and Larkin, 2018).

### Implications

The current study's examination of COVID-19's impact has provided some of the first evidence of preparatory beliefs and behaviors in the context of a global pandemic and psychosocial correlates. This study revealed that males and females have different prepping and coping responses during the pandemic, with males reporting higher rates of stockpiling. Additionally, stockpiling was related to psychopathology, catastrophic thinking, stress, anxiety, and traditionally male personality traits. These findings can be informative to developing future strategies for communities in responding to a global crisis. The knowledge that peers are considerably influential in driving behavior in a crisis can be utilized to suppress undesirable behaviors like panic buying. Additionally, public information can target cognitive factors that exacerbate competition and catastrophising.

The current pandemic has led to global increases in prepping behaviors like stockpiling. The current results suggest that stereotypical aspects of masculinity such as dominance, competitiveness, and exaggerated self-reliance is associated with stockpiling behaviors. These traits may be being nurtured in the current conditions of society as people put their own needs before others like we have observed throughout COVID-19 (Smith, 2005). Panic-buying may be ameliorated in environments that do not fuel these traditional hyper-masculine traits. An emphasis on togetherness may be a possible solution to encourage positive and self-aware behaviors. As group interactions are paramount in shaping behavior during crises (Abdulkareem et al., 2020), it may be productive to utilize these interactions and increase a sense of comradery to minimize self-serving behaviors like stockpiling.

The current findings have the potential to frame prepping as a dysfunctional and maladaptive coping response to stress and fear (Jurgens et al., 2019). These results show prepping was more related to personality traits, social learning, and doomsday interpretations than to levels of legitimate threat through proximity and vulnerability to the virus. Providing adequate public health guidance as well as mental health support during a crisis may aid in limiting hoarding behaviors. Furthermore, Strategies to address these psychological factors could be provided during ongoing waves of the pandemic or future crises.

### Strengths, Limitations, and Future Directions

The current study examined several unexplored areas in research thereby advancing our understanding of prepping characteristics. Previous research on prepping during pandemics is scarce and previous studies on doomsday prepping have failed to incorporate measures of psychopathology and rarely explore prepping in general samples. This study has also developed a new measure of prepping and coping during a pandemic (PCP-S) which has provided insights into the reasons for pandemic prepping and stockpiling, other ways of coping and gender differences.

This study also has a number of limitations. The new scale measuring prepping during the pandemic was developed rapidly to begin data collection during a rapidly evolving crisis, therefore further, studies are suggested to assess the psychometric properties of the scale, items and subscales. There were more female participants, although this was controlled for in multivariate analyses. Nevertheless, further studies are needed to explore and replicate the results. The majority of the participants were young psychology students from Australia. Because of the relatively small number of participants in other countries we were not able to make comparisons between countries. Additionally, participants self-selected to take part in the study, therefore they may not be representative of those who do not volunteer to participate in research. Further studies are needed to assess the generalizability of the results to broader populations. The finding regarding age would suggest that higher rates of prepping beliefs and behaviors may have been reported in an older sample. The relationship between age and prepping may be worth exploring further in future research.

The study's cross-sectional design does not allow for causal interpretations to be made. It is unclear if prepping beliefs and behaviors give rise to symptoms such as anxiety or if these variables increase the likelihood of prepping. Likewise, it may be the case that the spread of COVID-19 triggered an increase in prepping and simultaneously an increase in these symptoms. The ambiguity of the direction of these findings may be resolved by future longitudinal research.

## Conclusions

Overall, research on doomsday prepping is limited and more research is required to understand the striking increase in panic-buying and stockpiling during the COVID-19 pandemic. This study has provided some of the first evidence that preparatory beliefs and behaviors during the pandemic are related to anxiety, obsessive-compulsive-like symptoms, intolerance of uncertainty, and traditional masculinity traits. Additionally, males reported greater stockpiling of resources and prepping beliefs and behaviors than females. The study results suggest that stockpiling is fueled to a greater extent by seeing others stockpile, through a process of social learning, along with doomsday interpretations of COVID-19, than it is by actual proximity or vulnerability to infection. Overall, these findings indicate that doomsday prepping in the context of the current pandemic is grounded less in rational concerns than observational learning, as well as psychological and personality characteristics interacting with chaotic environments that lead to catastrophic thinking and feelings of fear and uncertainty.

## Data Availability Statement

The raw data supporting the conclusions of this article will be made available by the authors, without undue reservation.

## Ethics Statement

The studies involving human participants were reviewed and approved by Joint University of Wollongong and Illawarra Shoalhaven Local Health District Human Research Ethics Committee. The patients/participants provided their written informed consent to participate in this study.

## Author Contributions

NS had input into the study design, recruited participants, collected and analyzed data, and wrote the manuscript first draft. ST conceived the study, revised and edited the manuscript, oversaw the design, analyses, and interpretation of results. All authors agreed on the manuscript final content.

## Conflict of Interest

The authors declare that the research was conducted in the absence of any commercial or financial relationships that could be construed as a potential conflict of interest.

## References

[B1] Abdulkareem S. A. Augustijn E. Filatova T. Musial K. Mustafa Y. T. (2020). Risk perception and behavioral change during epidemics: comparing models of individual and collective learning. PLoS ONE 15:e0226483. 10.1371/journal.pone.0226483 31905206 PMC6944362

[B2] Antony M. M. Bieling P. J. Cox B. J. Enns M. W. Swinson R. P. (1998). Psychometric properties of the 42-item and 21-item versions of the depression anxiety stress scales in clinical groups and a community sample. Psychol. Assess. 10, 176–181. 10.1037/1040-3590.10.2.176

[B3] Apicella C. L. Dreber A. Gray P. B. Hoffman M. Little A. C. Campbell B. C. (2011). Androgens and competitiveness in men. J. Neurosci. Psychol. Econ. 4, 54–62. 10.1037/a0021979

[B4] Ballou S. Gray S. Palsson O. S. (2020). Validation of the pandemic emotional impact scale. Brain Behav. Immunity Health 9:100161. 10.1016/j.bbih.2020.100161 33103127 PMC7568491

[B5] Bandura A. (1977). Social Learning Theory, Vol. 1. Englewood Cliffs, NJ: Prentice-hall.

[B6] Boerma Y. E. de Boer M. M. van Balkom A. Eikelenboom M. Visser H. A. van Oppen P. (2019). Obsessive compulsive disorder with and without hoarding symptoms: characterizing differences. J. Affect. Disord. 246, 652–658. 10.1016/j.jad.2018.12.115 30611063

[B7] Carleton R. N. Norton P. J. Asmundson G. J. G. (2007). Fearing the unknown: a short version of the intolerance of uncertainty scale. J. Anxiety Disord. 21, 105–117. 10.1016/j.janxdis.2006.03.014 16647833

[B8] Carver C. S. (1997). You want to measure coping but your protocol's too long: consider the Brief COPE. Int. J. Behav. Med. 4, 92–100. 16250744 10.1207/s15327558ijbm0401_6

[B9] Cooper C. Katona C. Livingston G. (2008). Validity and reliability of the brief COPE in carers of people with dementia. J. Nerv. Ment. Dis. 196, 838–843. 10.1097/NMD.0b013e31818b504c 19008735

[B10] Feather N. T. McKee I. R. (2012). Values, right-wing authoritarianism, social dominance orientation, and ambivalent attitudes towards women. J. Appl. Soc. Psychol. 42, 2479–2504. 10.1111/j.1559-1816.2012.00950.x

[B11] Fetterman A. K. Rutjens B. T. Landkammer F. Wilkowski B. M. (2019). On post-apocalyptic and doomsday prepping beliefs: a new measure, its correlates, and the motivation to prep. Eur. J. Pers. 33, 506–525. 10.1002/per.2216

[B12] Foa E. B. Huppert J. D. Leiberg S. Langner R. Kichic R. Hajcak G.. (2002). The obsessive-compulsive inventory: development and validation of a short version. Psychol. Assess. 14, 485–496. 10.1037/1040-3590.14.4.485 12501574

[B13] Foels R. Pappas C. J. (2004). Learning and unlearning the myths we are taught: gender and social dominance orientation. Sex Roles 50, 743–757. 10.1023/B:SERS.0000029094.25107.d6

[B14] Fourtounas A. Thomas S. J. (2016). Cognitive factors predicting checking, procrastination and other maladaptive behaviours: prospective versus Inhibitory Intolerance of Uncertainty. J. Obsess. Compuls. Relat. Disord. 9, 30–35. 10.1016/j.jocrd.2016.02.003

[B15] Fung O. Loke A. (2010). Disaster preparedness of families with young children in Hong Kong. Scand. J. Public Health 38, 880–888. 10.1177/1403494810382477 20817655

[B16] Goodwin R. Gains S. O. Myers L. Neto F. (2011). Initial psychological responses to swine flu. Int. J. Behav. Med. 18, 88–92. 10.1007/s12529-010-9083-z 20195809 PMC7090401

[B17] Greenberg J. Solomon S. Pyszczynski T. (1997). Terror management theory of self-esteem and cultural world views: empirical assessments and conceptual refinements. Adv. Exp. Soc. Psychol. 29, 61–139. 10.1016/S0065-2601(08)60016-7

[B18] Hayes A. F. (2018). Introduction to Mediation, Moderation, and Conditional Process Analysis: A Regression-Based Approach, 2nd Edn. New York, NY: The Guilford Press.

[B19] Henry J. E. Crawford J. R. (2005). The short-form version of the depression anxiety stress scale (DASS-21): construct validity and normative data in a large non-clinical sample. Br. J. Clin. Psychol. 44, 227–239. 10.1348/014466505X29657 16004657

[B20] Horesh D. Brown A. D. (2020). Traumatic stress in the age of COVID-19: a call to close critical gaps and adapt to new realities. Psychol. Trauma Theory Res. Pract. Policy 12, 331–335. 10.1037/tra0000592 32271070

[B21] Jackson C. J. (2018). Are survivalists malevolent? Pers. Individ. Diff. 129, 104–107. 10.1016/j.paid.2018.03.006

[B22] Jacoby R. J. Fabricant L. E. Leonard R. C. Reimann B. C. Abramowitz J. S. (2013). Just to be certain: Confirming the factor structure of the Intolerance of Uncertainty Scale in patients with obsessive-compulsive disorder. J. Anxiety Disord. 27, 535–542. 10.1016/j.janxdis.2013.07.008 23973743

[B23] Jensen D. Cohen J. N. Mennin D. S. Fresco D. M. Heimberg R. G. (2016). Clarifying the unique associations among intolerance of uncertainty, anxiety, and depression. Cogn. Behav. Ther. 45, 431–444. 10.1080/16506073.2016.1197308 27314213 PMC5045801

[B24] Jonas E. McGregor I. Klackl J. Agroskin D. Fritsche I. Holbrook C.. (2014). Threat and defense: from anxiety to approach, in Advances in Experimental Social Psychology, Vol. 49, eds J. M. Olson and M. P. Zanna (Amsterdam: Elsevier Academic Press). p. 219–286

[B25] Jurgens C. Rupp C. Doebler P. Andor F. Buhlmann L. (2019). Metacognition in obsessive-compulsive disorder symptom dimensions: role of fusion beliefs, beliefs about rituals and stop signal. J. Obsess. Compuls. Relat. Disord. 21, 102–111. 10.1016/j.jocrd.2019.03.002

[B26] Kabel A. Chmidling C. (2014). Disaster prepper: health, identity, and american survivalist culture. Hum. Organ. 73, 258–266. 10.17730/humo.73.3.l34252tg03428527

[B27] Lovibond P. F. Lovibond S. H. (1995). The structure of negative emotional states: comparison of the depression anxiety stress scale (DASS) with the beck depression and anxiety inventories. Behav. Res. Ther. 33, 335–343. 10.1016/0005-7967(94)00075-U 7726811

[B28] Lowe B. Decker O. Muller S. Brahler E. Schellberg D. Herzog W.. (2008). Validation and standardization of the generalized anxiety disorder screener (GAD-7) in the general population. Med. Care 46, 266–274. 10.1097/MLR.0b013e318160d093 18388841

[B29] Matthews C. A. Delucchi K. Cath D. C. Willemsen G. Boomsma D. I. (2014). Partitioning the etiology of hoarding and obsessive-compulsive symptoms. Psychol. Med. 44, 2867–2876. 10.1017/S0033291714000269 25066062 PMC4429876

[B30] McEvoy P. A. Mahoney A. E. J. (2011). Achieving certainty about the structure of intolerance of uncertainty in a treatment-seeking sample with anxiety and depression. J. Anxiety Disord. 25, 112–122. 10.1016/j.janxdis.2010.08.010 20828984

[B31] Miranda R. Mennin D. S. (2004). Depression, generalised anxiety disorder, and certainty in pessimistic predictions about the future. Cogn. Ther. Res. 31, 71–82. 10.1007/s10608-006-9063-4

[B32] Morris N. Johnson M. P. (2002). Apocalyptic thinking, autonomy, and sociotropy. Psychol. Rep. 90, 1069–1074. 10.2466/pr0.2002.90.3c.1069 12150386

[B33] Routledge C. Abeyta A. A. Roylance C. (2018). Death and end times: the effects of religious fundamentalism and mortality salience on apocalyptic beliefs. Religion Brain Behav. 8, 21–30. 10.1080/2153599X.2016.1238840

[B34] Smith R. T. (2005). Characteristics of hypermasculinity: A relational perspective (Doctoral dissertation), Fielding Graduate University, Santa Barbara, CA, United States.

[B35] Snell W. E. (1989). Development and validation of the masculine behaviour scale: a measure of behaviours stereotypically attributed to males vs. females. Sex Roles 21, 749–767. 10.1007/BF00289806

[B36] Spence J. T. Helmreich R. L. (1978). Psychological Masculinity and Femininity. Austin: University of Texas Press.

[B37] Spitzer R. L. Kroenke K. Williams J. B. W. Lowe B. (2006). A brief measure for assessing generalised anxiety disorder. Arch. Intern. Med. 166, 1092–1097. 10.1001/archinte.166.10.1092 16717171

[B38] Tanovic E. Gee D. G. Joormann J. (2018). Intolerance of uncertainty: neural and psychophysiological correlates of the perception of uncertainty as threatening. Clin. Psychol. Rev. 60, 87–99. 10.1016/j.cpr.2018.01.001 29331446

[B39] Thomas S. Larkin T. (2018). Plasma cortisol and oxytocin levels predict help-seeking intentions for depressive symptoms. Psychoneuroendocrinology 87, 159–165. 10.1016/j.psyneuen.2017.10.018 29096223

[B40] Warrens M. J. (2015). Some relationships between Cronbach's alpha and the Spearman-Brown formula. J. Classific. 32, 127–137. 10.1007/s00357-015-9168-0

[B41] Wheaton M. G. Ward H. E. (2020). Intolerance of uncertainty and obsessive-compulsive personality disorder. Pers. Disord. Theory Res Treat. 11, 357–364. 10.1037/per0000396 32068417

[B42] World Health Organization (2020). WHO Director-General's Opening Remarks at the Media Briefing on COVID19 -March 2020. World Health Organization.

[B43] Yu H. Ho S. C. So K. Lo Y. L. (2005). Short communication: the psychological burden experienced by Hong Kong midlife women during the SARS epidemic. Stress Health 21, 177–184. 10.1002/smi.1051

[B44] Zheng R. Shou B. Yang J. (2020). Supply disruption management under consumer panic buying and social learning effects. Omega 101:102238. 10.1016/j.omega.2020.102238

