# Supplementary material for: Doomsday Prepping During the COVID-19 Pandemic
Source: Front Psychol. 2021 Apr 14;12:659925. doi: 10.3389/fpsyg.2021.659925 (PMC8079789; doi:10.3389/fpsyg.2021.659925)
Supplement: Supplementary file 1 [file Data_Sheet_1.docx]

**Supplementary Material**

**Prepping and Coping during a Pandemic Scale (PCP-Scale)**

1. In response to COVID-19 have you been stockpiling (obtaining more than usual) supplies such as toilet paper, non-perishable foods, hand sanitizer, etc.

not at all☐ somewhat☐ moderately so☐ very much so☐

1. If you have been stocking up on resources, to what extent have you been doing this for the following reasons? - Fears/concerns about supermarkets closing

not at all☐ somewhat☐ moderately so☐ very much so☐

1. If you have been stocking up on resources, to what extent have you been doing this for the following reasons? - Fears/ concerns about restrictions on going out or lockdowns

not at all☐ somewhat☐ moderately so☐ very much so☐

1. If you have been stocking up on resources, to what extent have you been doing this for the following reasons? - In response to other people buying excessive amounts of supplies

not at all☐ somewhat☐ moderately so☐ very much so☐

1. If you have been stocking up on resources, to what extent have you been doing this for the following reasons? - Because friends or family have been advising me to

not at all☐ somewhat☐ moderately so☐ very much so☐

1. If you have been stocking up on resources, to what extent have you been doing this for the following reasons? - Fears/concerns about getting sick and not being able to go out

not at all☐ somewhat☐ moderately so☐ very much so☐

1. To what extent do you associate the current COVID-19 situation with a "doomsday" scenario?

not at all☐ somewhat☐ moderately so☐ very much so☐

1. Does the current situation of COVID-19 make you worry about the future of society?

not at all☐ somewhat☐ moderately so☐ very much so☐

1. During this time to what extent have you been doing any of the following to remain positive? - Socialising with friends and family via technology

not at all☐ somewhat☐ moderately so☐ very much so☐

1. During this time to what extent have you been doing any of the following to remain positive? - Reading or engaging in support forums

not at all☐ somewhat☐ moderately so☐ very much so☐

1. During this time to what extent have you been doing any of the following to remain positive? - Focusing on personal interests/ goals/ hobbies

not at all☐ somewhat☐ moderately so☐ very much so☐

1. During this time to what extent have you been doing any of the following to remain positive? – Exercise

not at all☐ somewhat☐ moderately so☐ very much so☐

1. During this time to what extent have you been doing any of the following to remain positive? - Practicing meditation/mindfulness

not at all☐ somewhat☐ moderately so☐ very much so☐

1. During this time to what extent have you been doing any of the following to remain positive? - Engaging in spiritual practice or thought

not at all☐ somewhat☐ moderately so☐ very much so☐

1. During this time to what extent have you been doing any of the following to remain positive? - Undertaking home maintenance/improvements

not at all☐ somewhat☐ moderately so☐ very much so☐

1. During this time to what extent have you been doing any of the following to remain positive? - Spending time with pets

not at all☐ somewhat☐ moderately so☐ very much so☐

1. During this time to what extent have you been doing any of the following to remain positive? - Looking for any positives that have emerged due to the situation. E.g. reductions in pollution, the return of animals to certain areas, etc.

not at all☐ somewhat☐ moderately so☐ very much so☐

1. During this time to what extent have you been doing any of the following to remain positive? - Consulting a doctor

not at all☐ somewhat☐ moderately so☐ very much so☐

1. During this time to what extent have you been doing any of the following to remain positive? - Consulting a psychologist

not at all☐ somewhat☐ moderately so☐ very much so☐

1. During this time to what extent have you been doing any of the following to remain positive? - Phoning a helpline

not at all☐ somewhat☐ moderately so☐ very much so☐

**Contextual (control) questions**

1. Are there many confirmed cases of COVID-19 in your city?

not at all☐ somewhat☐ moderately so☐ very much so☐

1. Would you be considered as someone who is vulnerable to contracting COVID-19? E.g. elderly, compromised immune system, asthmatic, etc.

not at all☐ somewhat☐ moderately so☐ very much so☐

1. To what extent are you currently preparing or previously prepared for any of the following? - COVID-19

not at all☐ somewhat☐ moderately so☐ very much so☐

1. To what extent are you currently preparing or previously prepared for any of the following? - Other contagious health concerns

not at all☐ somewhat☐ moderately so☐ very much so☐

1. To what extent are you currently preparing or previously prepared for any of the following? - Natural disaster

not at all☐ somewhat☐ moderately so☐ very much so☐

1. To what extent are you currently preparing or previously prepared for any of the following? - Terrorism attack

not at all☐ somewhat☐ moderately so☐ very much so☐

1. To what extent are you currently preparing or previously prepared for any of the following? – War

not at all☐ somewhat☐ moderately so☐ very much so☐

1. To what extent are you currently preparing or previously prepared for any of the following? - Financial collapse

not at all☐ somewhat☐ moderately so☐ very much so☐

1. To what extent are you currently preparing or previously prepared for any of the following? - Political collapse

not at all☐ somewhat☐ moderately so☐ very much so☐

1. To what extent are you currently preparing or previously prepared for any of the following? - Climate change

not at all☐ somewhat☐ moderately so☐ very much so☐

1. Have you previously experienced or been personally impacted (i.e. a change to your personal, daily life) by any of the following? - Natural Disaster

not at all☐ somewhat☐ moderately so☐ very much so☐

1. Have you previously experienced or been personally impacted (i.e. a change to your personal, daily life) by any of the following? - Terrorism Attack

not at all☐ somewhat☐ moderately so☐ very much so☐

1. Have you previously experienced or been personally impacted (i.e. a change to your personal, daily life) by any of the following? - Economic Downturn

not at all☐ somewhat☐ moderately so☐ very much so☐

1. Have you previously experienced or been personally impacted (i.e. a change to your personal, daily life) by any of the following? - Health Pandemic

not at all☐ somewhat☐ moderately so☐ very much so☐

1. Have you previously experienced or been personally impacted (i.e. a change to your personal, daily life) by any of the following? – War

not at all☐ somewhat☐ moderately so☐ very much so☐

**Open/free text questions**

1. Are you currently preparing or previously prepared for anything not listed in the previous question? (If yes, please specify)

______________________________________________________________________________

1. If you have been stocking up on resources, have you been doing this for any reasons not stated in the previous question? (please explain)

______________________________________________________________________________

1. Have you been doing anything to remain positive that was not mentioned in the previous question?

______________________________________________________________________________
